# Gender differences in physical activity and sedentary behavior: Results from over 200,000 Latin-American children and adolescents

**DOI:** 10.1371/journal.pone.0255353

**Published:** 2021-08-12

**Authors:** Javier Brazo-Sayavera, Salomé Aubert, Joel D. Barnes, Silvia A. González, Mark S. Tremblay

**Affiliations:** 1 PDU EFISAL, Centro Universitario Regional Noreste, Universidad de la República, Rivera, Uruguay; 2 Healthy Active Living and Obesity Research Group, CHEO Research Institute, Ottawa, Ontario, Canada; La Inmaculada Teacher Training Centre (University of Granada), SPAIN

## Abstract

More physical activity and less sedentary behavior is beneficial for children and adolescents. Worldwide, gender differences are >8% favorable for men and the Latin-American region presents an even higher level of insufficient physical activity among women, with a lack of information in young population. Thus, the aim of the current study was to describe the gender differences in physical activity and recreational sedentary behavior in children and adolescents from Latin-American countries. The targeted age range was 5 to 17 years and included 219,803 participants (106,698 boys and 113,105 girls) from 33 out of 47 Latin-American countries identified. Physical activity guidelines from the World Health Organization (≥60 minutes of moderate-to-vigorous physical activity seven days of the week) and <3 hours recreational sedentary behavior daily were the references. In general, boys showed a higher prevalence of meeting physical activity guidelines in comparison with girls. A higher proportion of girls met the <3 hours recreational sedentary behavior cut-point in only ten countries. Thirty percent of the identified countries had no available data. The majority had data from the Global School-based Student Health Survey with data principally on adolescents and only 11/33 countries reported data in the last 5-year period. In conclusion, gender differences in the compliance with physical activity guidelines and the <3 hours recreational sedentary behavior cut-point are evident among children and adolescents from Latin-American countries, with boys being more active than girls.

## Introduction

Approximately a quarter of the general population is estimated to meet the global recommendations for physical activity (PA) [1], with a difference of >8% by gender in favor of men [2]. Similar data have not been published regarding sedentary behaviors (SB).

Latin American adults present the highest prevalence of insufficient PA (39.1%) in comparison with other areas of the world [2]. In particular, Latin-American women were identified as the adult group with the highest level of insufficient PA in the world (43.7%) and the gap between males and females is up to 9.4 percentage points [2]. This difference between genders has been confirmed in a more recent study across six Latin-American countries, where notable gender differences have been reported [3]. Equity, in terms of achieving PA guidelines, has been studied previously in different countries from South America. Azevedo et al. (2007) [4] studied differences in leisure time PA in the Brazilian population and found that men had higher levels of PA than women. In Uruguay, young male adults were more than twice as likely to meet PA guidelines (72.8%) in comparison with young female adults (27.2%) [5]. Moreover, Colombian males were found more active than their female counterparts, and socioeconomic status was identified as an important factor to explain the observed inequalities [6]. With regard to SB in Latin-America, there were no reported gender differences in sitting time in adults among the analyzed countries (Argentina, Chile, Ecuador, Peru and Surinam) with the exception of Brazil [3].

In the past decade, interest in studying gender differences in movement behaviors at younger ages has emerged, with the intention to strive for a gender-equitable society. Recently, a study including 36 countries around the world concluded that gender inequality exists in adolescents PA [7]. Guthold, Stevens, Riley, & Bull (2020) [8] have presented data from the Global School-based Health Survey (GSHS) from 146 countries around the world (32 countries or overseas colonies in Latin-America and Caribbean), showing the prevalence and trends of insufficient PA among adolescents. The Latin American region had a global prevalence of insufficient PA of 84.3%, being in the middle in comparison with other regions. The gender gap was 9% favorable to male adolescents [8]. Similarly, Aguilar-Farias et al. (2018) [9] studied PA among adolescents from Latin-American and Caribbean countries, finding gender disparities in several countries. However, all of these studies focused only on adolescents, highlighting the need to study what happens in the whole age range of children and adolescents (5–17 years).

In response to the international need for healthy movement behavior surveillance and advocacy in children and youth, the Active Healthy Kids Global Alliance Global Matrix studied the behavioral indicators related with PA [10]. However, it has not yet focused on gender inequalities. Among the Latin-American countries participating in the Global Matrix project (Brazil, Chile, Colombia, Ecuador, Mexico, Venezuela and Uruguay), only Mexico reported the prevalence meeting PA guidelines separately between genders, with a higher prevalence in boys (21.8%) than in girls (12.7%) [11]. None of these countries reported SB results separately by gender.

PA provides several health benefits for children and youth and evidence indicates that the more PA, the greater these benefits [12]. However, it is unclear whether the benefits are the same by gender because the physiological response differs between boys and girls, with the type and intensity of the physical activities chosen as main factors in this response [13]. In addition, SB is known as a separate risk factor for cardiometabolic disease and all-cause mortality [14]. Men and women may engage in different SB, and therefore, potentially face different health consequences [15]. Thus, there is a need for detailed information on PA and SB by gender for the development of tailored healthy active lifestyle promotion actions and policies. Taking into account that the data reported to date is mainly focused on adults and adolescents, the aim of the present study was to describe the gender differences in PA and SB in children and adolescents from Latin-American countries.

## Methods

### Data sources and participants

The targeted age range was 5 to 17 years, aligning with the age range of the Global Matrix [10]. Territories (countries and dependent territories) from Latin-America and the Caribbean were identified for the current study following the World Bank classification. The main selection criterion was to be a country or a dependent territory in Latin-America and the Caribbean; therefore 47 different territories were identified for the current study: Anguilla, Antigua and Barbuda, Argentina, Aruba, Bahamas, Barbados, Belize, Bermuda, Bolivia, Brazil, British Virgin Islands, Cayman Islands, Chile, Colombia, Costa Rica, Cuba, Curaçao, Dominica, Dominican Republic, Ecuador, El Salvador, French Guiana, Grenada, Guadalupe, Guatemala, Guyana, Haiti, Honduras, Jamaica, Martinique, Mexico, Montserrat, Netherland Antilles, Nicaragua, Panama, Paraguay, Peru, Puerto Rico, Saint Kitts and Nevis, Saint Lucia, Saint Vincent and Grenadines, Sint Maarten, Suriname, Trinidad and Tobago, Turks and Caicos, Uruguay and Venezuela.

Multiple sources of information were included to gather as much information as possible. For the countries that participated in the Global Matrix 3.0 (Brazil, Chile, Colombia, Ecuador, Mexico, Uruguay, Venezuela), the main sources of information were already identified [11, 16–21]. For the remaining countries, a first contact was done through email to the GSHS report contact for each country [22]. Subsequently, government public health institutions were contacted for the countries without GSHS contacts and for the countries who did not respond to the email sent in the previous step to identify relevant source of information.

**Fig 1:**
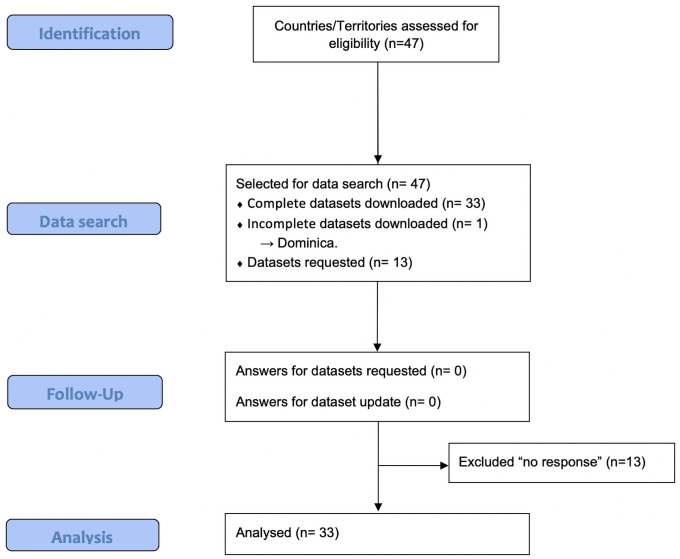
Flowchart of procedures for data acquisition.

Most of the data were extracted from the raw data provided by the GSHS reported by the World Health Organization [22]. This survey has a standardized sample technique and study protocol, using the same questionnaire for all participating countries. Some of the countries selected for the present study participated in more than one survey between 2006 and 2017 and in this case the more recent dataset was selected for analysis. Bolivia (only for PA), Brazil, Colombia, Ecuador and Mexico had data from other nationally representative surveys that were also included. Dominica did not include all the data necessary for the analysis (only data for PA analysis). Finally, several countries (Aruba, Bermuda, Cuba, French Guiana, Guadalupe, Haiti, Martinique, Netherland Antilles, Nicaragua, Panama, Puerto Rico, Sint Maarten, Turks and Caicos and Venezuela) were excluded after several unsuccessful attempts to find a local contact or source of information. A flow diagram presenting details about the datasets included is shown in Fig 1.

### Outcomes

Data on the compliance with PA guidelines and recreations SB cut-points were extracted by gender and age from available datasets. GSHS data were extracted from the following question: “*During the past 7 days*, *on how many days were you physically active for a total of at least 60 minutes per day*?*”*, and the reply “*7 days”* was the identified criteria for meeting the WHO PA guidelines [23]. Data about time spent in SB daily, assessed principally based on leisure screen time, were also extracted. Less than three hours of recreational sedentary time daily was the GSHS cut-point for SB and was selected as the cut-point for our analysis, following previous studies [24]. The questions used in the GSHS and the other included surveys are presented in Table 1.

**Table 1:** Questions used to assess physical activity and recreational sedentary behavior in the different surveys included.

Countries	Physical activity assessment	Recreational sedentary behavior assessment
**GSHS Countries**	During the past 7 days, on how many days were you physically active for a total of at least 60 minutes per day?	How much time do you spend during a typical or usual day sitting and watching television, playing computer games, talking with friends or doing other sitting activities? Each country could add specific sedentary activities at the end of the question.
**Bolivia** National Household Survey	In your leisure time, do you practice any physical activity or sport during at least 30 consecutive minutes? (i.e., running, riding a bike, doing exercise at gym or at home…) In a typical week, how many days do you do this activity?	How much time do you spend during a typical or usual day sitting and watching television, playing computer games, talking with friends or doing other sitting activities?
**Brazil** National Survey on School Health	During the past 7 days, on how many days were you physically active for a total of at least 60 minutes (1 hour) per day? (Principally the time you spent in any type of physical activity in each day)	In a normal day, how much time do you spent sitting, watching TV, using computer, playing videogames, chatting with friends or doing other activities sitting? (Do not taking into account Saturdays, Sundays, Holidays and the time sitting at school)
**Colombia** National Survey on the Nutritional Status	During the last week (past 7 days), on how many days were you physically active for at least 60 minutes per day? (Add up all the time you spent in any kind of physical activity that increased your heart rate and made you breathe hard some of the time)	On an average school day or weekday, how many hours did you watch TV? On an average school day or weekday, how many hours did you play video games or use a computer for something that is not school work? (Count time spent using Xbox, PlayStation, iPod, iPad or other tablet, smartphone, cellphone, YouTube, Facebook or other social media and internet.)
**Ecuador** National Survey on Health and Nutrition	During the last 7 days, were you active during at least 60 consecutive minutes every day? Take into account moderate activities as walking, riding a bike, playing outdoors as well as intense activities as playing football, volleyball or running.	During the last 7 days, did you watch TV and/or play videogames (without corporal movement or physical activity)? How many days did you watch TV and/or play videogames without movement (without corporal movement or physical activity)? Please, provide the days and the time in which you watched TV and/or played videogames that did not include corporal movement?
**Mexico** National Survey on Health and Nutrition	In the last 7 days, how many days were active during at least 60 consecutive minutes every day?	The total sum of activities in front of a screen including weekday and weekend day “How many hours do you spend in front of a screen, watching TV, playing videogames or with a computer, electronic tablet or mobile phone? Add time during the morning, afternoon and evening.

### Statistical analysis

Data from countries were pooled into a single dataset for analysis. Generalized linear models were fitted for each country to estimate the odds of boys meeting PA guidelines and the recreational SB cut-point compared to girls. Data were adjusted for age. All analyses were performed in RStudio 1.3.1056.

## Results

Table 2 shows the sources of information and characteristics of the included countries. Of the 33 included countries, the majority (n = 28, 85%) had data from the GSHS and only five had other data from nationally representative surveys. Ten countries had information from 2010 or earlier, another twelve countries had data before 2015 and the rest (n = 11) had data from the last 5 years. The response rate was over 70% except in a couple of countries (Antigua and Barbuda and Chile) and other countries with national surveys did not provide the response rate. The age range was between 5 and 17 years, but mostly concentrated within the range of 11 to 17 years. There were more girls (n = 113,105) than boys (n = 106,698) in the final analytical sample.

**Table 2:** Descriptive characteristics of the surveys included in the study.

Country	Survey name	Survey year	Response rate	Age range	Sample size	% Boys	Missing data PA	Missing data SB
Anguilla	GSHS	2016	88%	12–17	807	47.3	30	32
Antigua and Barbuda	GSHS	2009	67%	11–16	1,258	45.2	32	75
Argentina	GSHS	2012	71%	11–16	28,134	47.2	803	1,051
Bahamas	GSHS	2013	78%	11–17	1,353	46.3	41	73
Barbados	GSHS	2011	73%	11–16	1,626	45.1	68	32
Belize	GSHS	2011	88%	11–16	2,091	47.1	84	57
Bolivia	GSHS-NHS	2012–2018	88%-N/A	5–17	3,544–9,749	49.5–51.8	47–6,044	68–9,749
Brazil	PeNSE	2015	N/A	11–17	116,280	48.4	654	296
British Virgin Islands	GSHS	2009	90%	11–16	1,652	44.2	20	43
Cayman Islands	GSHS	2007	79%	11–16	1,297	48.3	68	114
Chile	GSHS	2013	60%	11–17	1,902	48.8	11	66
Colombia	ENSIN	2015	N/A	6–17	12,105	51.4	8	149
Costa Rica	GSHS	2009	72%	11–16	2,667	48.1	16	22
Curaçao	GSHS	2015	86%	11–17	2,234	45.5	118	124
Dominica	GSHS	2009	84%	11–16	1,635	43.3	108	1,635
Dominican Republic	GSHS	2016	74%	11–17	1,374	43.0	39	41
Ecuador	ENSANUT	2012	N/A	5–17	28,977	50.1	21,430	21,430
El Salvador	GSHS	2013	88%	11–16	1,886	53.2	14	26
Grenada	GSHS	2008	82%	11–16	1,531	44.5	77	94
Guatemala	GSHS	2015	85%	11–17	4,264	48.9	146	293
Guyana	GSHS	2010	76%	11–16	2,380	43.4	51	42
Honduras	GSHS	2012	79%	11–16	1,763	47.4	38	37
Jamaica	GSHS	2017	71%	11–17	1,612	45.3	54	67
México	ENSANUT	2016	N/A	10–17	2,800	47.4	48	8
Montserrat	GSHS	2008	78%	11–17	211	47.4	4	11
Paraguay	GSHS	2017	87%	11–17	2,979	47.0	77	51
Peru	GSHS	2011	85%	11–16	2,867	48.7	15	5
Saint Kitts and Nevis	GSHS	2011	70%	11–16	1,730	43.8	28	36
Saint Lucia	GSHS	2007	82%	11–16	1,273	42.3	19	19
Saint Vincent and Grenadines	GSHS	2007	84%	11–16	1,309	46.6	73	80
Suriname	GSHS	2016	83%	11–17	1,913	48.3	27	18
Trinidad and Tobago	GSHS	2017	89%	11–17	3,784	46.3	164	168
Uruguay	GSHS	2012	77%	11–16	3,488	46.2	26	58

GSHS: Global School-based Student Health Survey; NHS: National Health Survey from Bolivia; PeNSE: National Adolescent School-based Health Survey from Brazil; ENSIN: National Nutrition Status Survey from Colombia; ENSANUT: National Health and Nutrition Survey (Ecuador and Mexico); N/A: Not available; PA: Physical activity; SB: Sedentary behavior; Missing data: Data from the total sample not included in the analysis.

**Fig 2:**
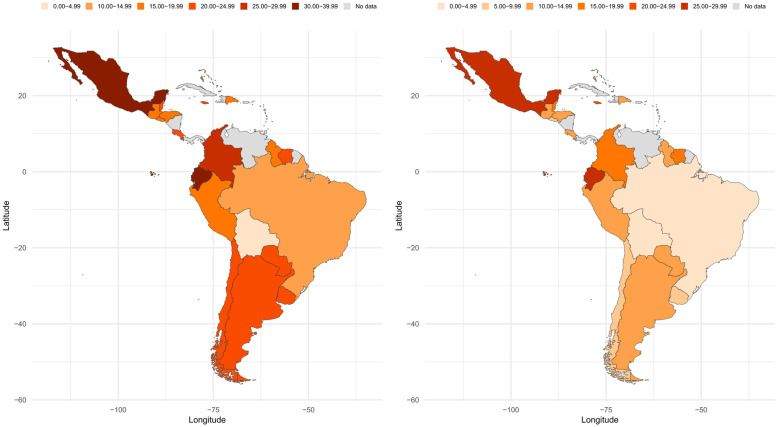
Prevalence map of meeting 2010 WHO PA guidelines among Latin-American children and adolescents. Left: Boys; Right: Girls. These maps were rendered in RStudio 1.4.1103 using the rworldmap [25] and ggplot2 [26] R packages.

Prevalence of meeting PA guidelines is presented in the Fig 2 (S1 Table contains the calculated prevalence with 95% confidence intervals). In general, boys showed a higher prevalence of meeting PA guidelines in comparison with girls. The range in boys was between 4.8% (Bolivia) and 37.8% (Mexico), while the range in girls was between 3.5% (Bolivia) and 26.2% (Ecuador). The greatest difference between boys and girls was observed in Uruguay (13.5%) and the lowest in Jamaica (1.8%).

**Fig 3:**
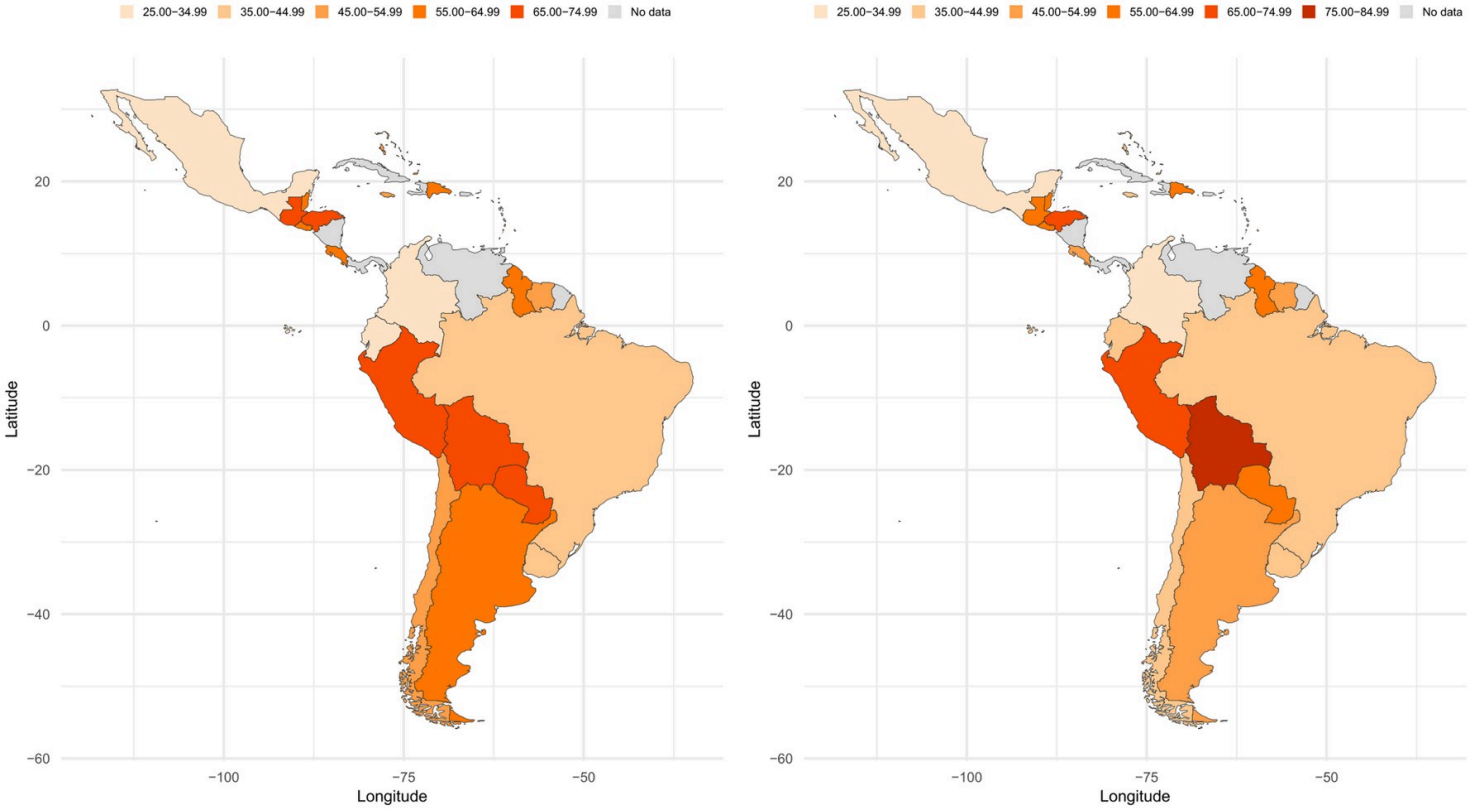
Prevalence of meeting the <3 hours recreational SB cut-point among Latin American children and adolescents. Left: Boys; Right: Girls. These maps were rendered in RStudio 1.4.1103 using the rworldmap [25] and ggplot2 [26] R packages.

Results for meeting the <3 hours recreational SB cut-point are provided in Fig 3. The ranges of prevalence of meeting the cut-point between boys and girls were similar: from 26.8% (Mexico) to 70.7% (Peru) for boys and 27.6% (Mexico) to 76.4% (Bolivia) in girls. The highest difference between boys and girls was in Anguilla (13.1%) and the lowest in Grenada (<1.0%).

**Fig 4:**
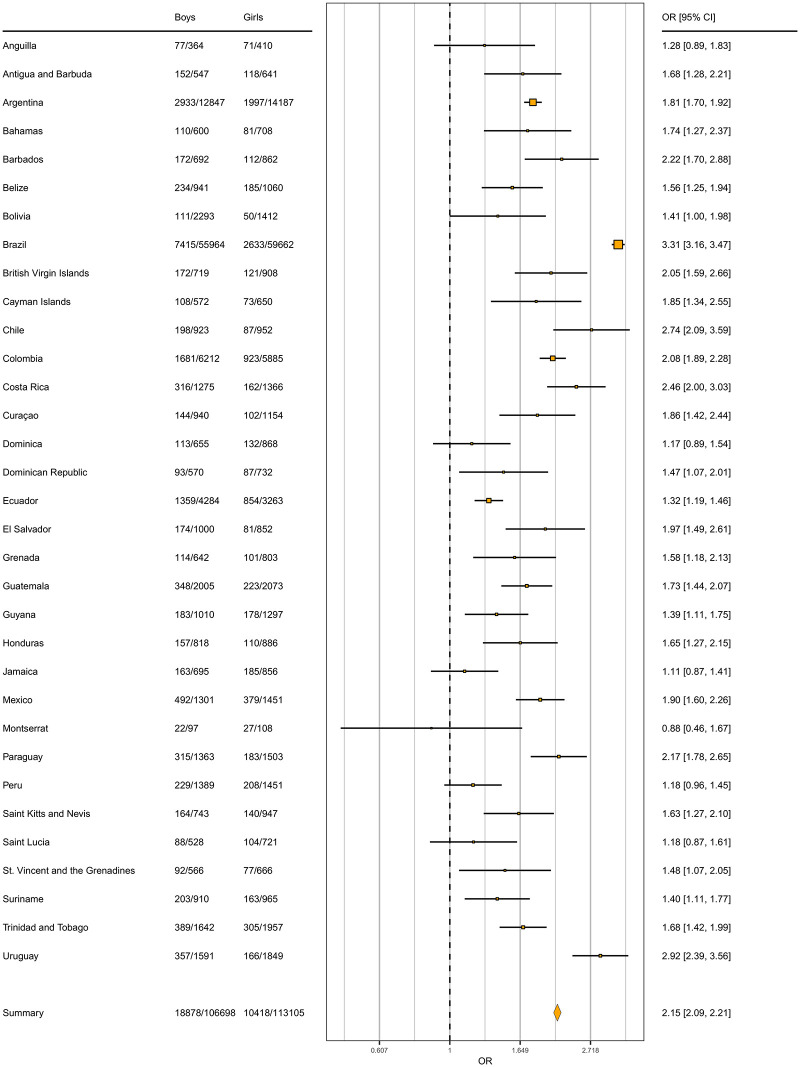
Forest plot showing the odds ratios of meeting international PA guidelines by gender in children and adolescents from Latin-America, by country. Reference = girl.

The average odds ratio of meeting PA guidelines for children and adolescents in Latin-America was 2.15 (Fig 4) in favor of boys. Brazil and Uruguay were the countries with the highest odds ratio for boys and girls meeting PA guidelines (3.31 and 2.92, respectively). Montserrat presented the lowest overall odds ratio with 0.88, followed by Jamaica (1.11).

**Fig 5:**
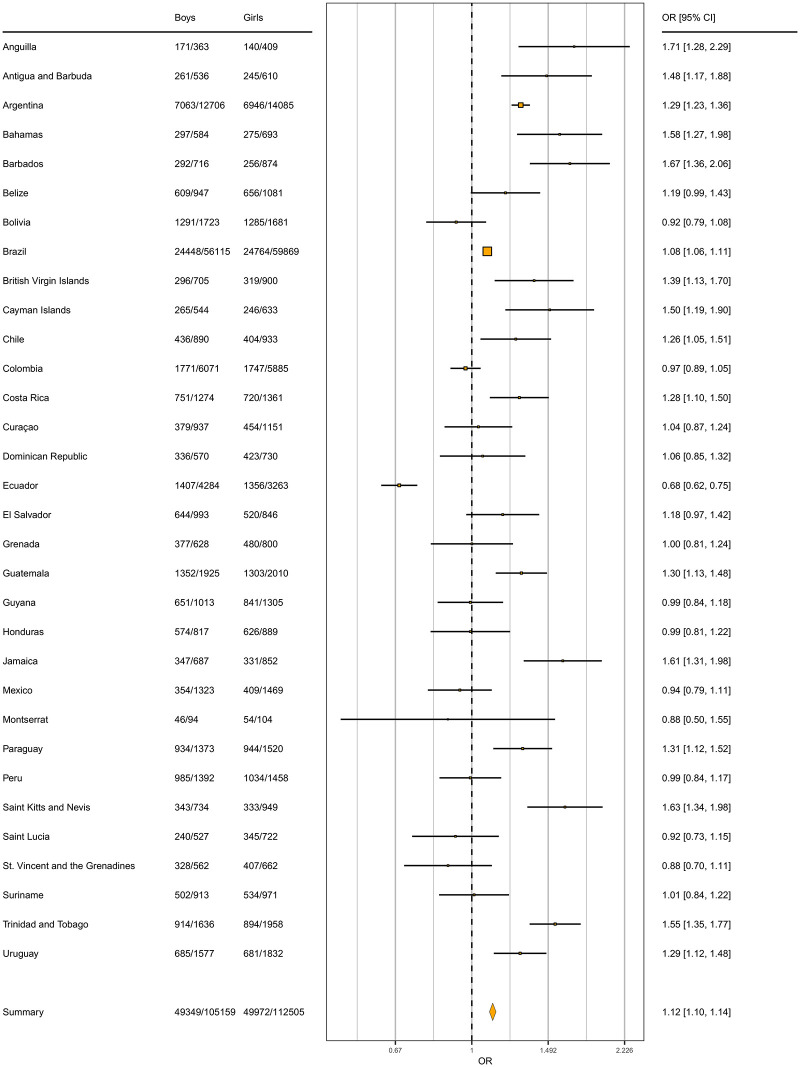
Forest plot showing the odds ratio of meeting the <3 hours recreational SB cut-point by gender in children and adolescents from Latin-America, by country. Reference = girl.

For SB, the overall odds ratio of getting <3 hours of daily recreational sedentary behavior for children and youth in Latin-America was 1.12 (Fig 5), again in favor of boys. Dominica did not report data on SB. Anguilla and Barbados had the highest odds ratio between boys and girls (1.71 and 1.67, respectively), while Ecuador followed by Saint Vincent and the Grenadines and Montserrat had the lowest odds ratio with 0.68 and 0.88, respectively.

## Discussion

Large gender disparities in terms of prevalence of meeting PA guidelines and the recreational SB cut-point were observed among children and adolescents across Latin-American countries.

The present results are in accordance with recent studies showing PA gender differences among adolescents in the region of the Americas [2, 9, 27]. Overall, in Latin-America, boys are more likely to meet the PA guidelines than girls (OR = 2.15 on average). Gender inequities were especially concerning in six countries (Barbados, Brazil, Chile, Costa Rica, Paraguay and Uruguay) with ORs higher than the average. Previous studies [28] around the world have reported PA differences between genders and these differences have been attributed to lower participation of girls in organized sports [29] as well as socio-ecological factors at the individual (e.g., body weight, fitness, boy’s preferences for higher intensity activities or perceived competence), family (e.g., parent’s support, gender roles, living conditions or family structure), community (e.g., participation in community sport), school (e.g., opportunities for students to be physically active during school lunch breaks) and environmental level (e.g., climate or geography) [30]. Montserrat was the only territory where girls were estimated to be more active than boys. However, this finding was based on a survey with a small number of participants (n = 205) in comparison with the rest of analyzed countries. To the best of our knowledge, there is not a specific public policy promoting physical activity in Montserrat [31]. The small population and the higher proportion of women in Montserrat could potentially be responsible for this result favorable to girls [32].

SB was analyzed mostly based on the assessment of recreational sedentary time. Overall, boys were more likely to meet the recreational SB cut-point than girls (OR 1.12 on average) in accordance with previous results from other countries [33]. In addition, in ten countries (Bolivia, Colombia, Ecuador, Guyana, Honduras, Mexico, Montserrat, Peru, Saint Lucia, and St. Vincent and the Granadines), girls were more likely to meet the recreational SB cut-point than boys. These differences could potentially be related to the gender difference in the preference of leisure activities as it has been observed in the study by Taverno Ross et al. (2013) [34]. They found that the preferred activities for girls were listening to music or texting or talking on the phone, while boys spent more time playing video games. Further research is needed to understand the drivers of these disparities, since there is a lack of study from Latin-America exploring the factors that could potentially explain the observed gender disparities in recreational sedentary behavior time among children and adolescent in our present analysis [9].

### Strengths and limitations

The GSHS was the major source of the data included in our analysis and it was collected using self-reported questionnaires and some of the included data date back to 2007. High-income countries (e.g., USA with the NHANES [35]) often monitor these behaviors using additional methods (i.e., accelerometry) in subsamples. However, even though the use of self-reported data is widely accepted for PA and SB surveillance, this methodology reports more vigorous PA and less sedentary time compared with the accelerometers [36]. In addition, the GSHS focused mostly on adolescents between 13 and 17 years, even though the datasets contain data from younger adolescents too. Some of the included dataset also came from national surveys including various age ranges and sample sizes. The PA and SB assessment methods were not consistent across all the data included in the current study and were collected with surveys using different questionnaires. Furthermore, these questionnaires presented several limitations: it was unclear in some of the questionnaires if school time PA should be included, resulting in the participants potentially excluding it in their report; and not all of these questionnaires have been validated against an objective measurement method or in the particular language/country context it was used. Moreover, some of the included datasets did not allow to assess the proportion of children meeting the <3 hours recreational SB cut-point from the GSHS. In addition, there is a lack of information about the bout duration, which could provide further information about the differences in recreational sedentary behavior between boys and girls.

Nevertheless, this is the first study providing population-level information on gender differences in meeting PA guidelines and recreational SB cut-point in Latin-American children and adolescents based on the compilation of the best available data, identified by a local contact within most included countries. The current study also highlights that the needs in terms of PA and SB promotion are different by gender and by country across Latin-America.

### Research gaps and future directions

The current study identifies the paucity of recent data as several countries are still lacking surveillance of PA and SB and/or some of the most recent available datasets were more than 10 years old. Approximately 30% of the countries included in this analysis had no public or available data and only 11/33 countries had available data that was collected in the last 5 years. Furthermore, the accessibility of existing data was also an issue encountered in the development of the current study. Providing a rapid, open and transparent access to public health data should become a priority across Latin-America to support the improvement of research and the development of adapted efficient health strategies and policies as well as to provide the opportunity of surveillance for preventing non-communicable diseases [37].

With the publication of new global PA and SB guidelines [1], the current analysis could change if the new PA guidelines were employed for active children and adolescents (“at least an average of 60 min/day of moderate- to vigorous-intensity, mostly aerobic, PA across the week”) in comparison to the previous statement (“accumulate at least 60 minutes of MVPA per day”). Therefore, we recommend that future surveillance initiatives should assess the prevalence of meeting the new guidelines to assess PA among children and adolescents at the population level.

This study highlighted the need for the development of a standardized, validated and internationally adopted PA and SB surveillance tool across Latin-America. An improved methodology common for all the Latin-American countries would be essential to study rigorously the differences in meeting PA and SB guidelines among children and adolescents and perform international comparisons, in particular to take into account the new WHO PA and SB guidelines [1]. Currently, to our knowledge, the only multi-country tool validated in some South-American countries is SAYCARE [38, 39].

Finally, the findings presented in this study contribute to concerns about the level of PA and SB among children and adolescents across Latin-America, in particular among girls. Further research is needed to identify the determinants of the gender differences observed across Latin-American countries for the development of strategic and efficient healthy active lifestyle promotion actions and policies.

## Conclusion

Gender differences in meeting PA and SB recommendations are evident among children and adolescents from Latin-American countries, where boys are more active than girls. For SB, boys were generally more likely to meet a limit of 3 hours of recreational sedentary activities, however the gap with girls is lower in comparison with PA.

The lack of good quality or current surveillance data capturing gender differences in PA and SB was a major limitation for the study. Significant work is needed to improve the surveillance systems for child and adolescent PA and SB with the development of a standardized, validated and internationally adopted assessment tool across Latin-America, taking into account the new PA recommendations [1].

## Supporting information

S1 Table Prevalence of meeting PA guidelines and <3 hours recreational SB cut-point by gender and country. (DOCX)
