# Supplementary material for: Gender differences in physical activity and sedentary behavior: Results from over 200,000 Latin-American children and adolescents
Source: PLoS One. 2021 Aug 12;16(8):e0255353. doi: 10.1371/journal.pone.0255353 (PMC8360534; doi:10.1371/journal.pone.0255353)
Supplement: S1 Table — (DOCX) [file pone.0255353.s001.docx]

**S1 Table.** Prevalence of meeting PA guidelines and SB cut-point by gender and country.

| **Country** | **Prevalence PA boys** | **Prevalence PA girls** | **Prevalence SB boys** | **Prevalence SB girls** |
| --- | --- | --- | --- | --- |
| Anguilla | 21.2 (17.1 - 25.8) | 17.3 (13.9 - 21.4) | 46.9 (41.7 - 52.2) | 33.8 (29.3 - 38.7) |
| Antigua and Barbuda | 27.8 (24.1 - 31.8) | 18.4 (15.5 - 21.7) | 48.8 (44.4 - 53.1) | 40.3 (36.4 - 44.3) |
| Argentina | 22.8 (22.1 - 23.6) | 14.1 (13.5 - 14.7) | 55.6 (54.7 - 56.4) | 49.2 (48.4 - 50.1) |
| Bahamas | 18.3 (15.4 - 21.7) | 11.4 (9.2 - 14.1) | 51.0 (46.8 - 55.1) | 39.8 (36.1 - 43.6) |
| Barbados | 24.9 (21.7 - 28.3) | 13.0 (10.9 - 15.5) | 40.3 (36.6 - 44.1) | 29.0 (26.0 - 32.2) |
| Belize | 24.9 (22.2 - 27.8) | 17.5 (15.2 - 19.9) | 63.9 (60.7 - 67.0) | 60.5 (57.4 - 63.4) |
| Bolivia | 4.8 (4.0 - 5.8) | 3.5 (2.7 - 4.7) | 74.9 (72.7 - 76.9) | 76.4 (74.3 - 78.4) |
| Brazil | 13.2 (13.0 - 13.5) | 4.4 (4.3 - 4.6) | 43.6 (43.2 - 44.0) | 41.4 (41.0 - 41.8) |
| British Virgin Islands | 23.9 (20.9 - 27.2) | 13.3 (11.2 - 15.8) | 41.8 (38.1 - 45.6) | 35.4 (32.3 - 38.6) |
| Cayman Islands | 18.9 (15.8 - 22.4) | 11.2 (9.0 - 14.0) | 48.1 (43.9 - 52.5) | 38.9 (35.1 - 42.8) |
| Chile | 21.5 (18.9 - 24.3) | 9.1 (7.4 - 11.2) | 49.0 (45.6 - 52.3) | 43.3 (40.1 - 46.6) |
| Colombia | 27.1 (26.0 - 28.2) | 15.7 (14.8 - 16.6) | 29.2 (28.0 - 30.3) | 29.7 (28.5 - 30.9) |
| Costa Rica | 24.8 (22.5 - 27.3) | 11.9 (10.2 - 13.7) | 59.1 (56.3 - 61.8) | 52.9 (50.2 - 55.6) |
| Curaçao | 15.3 (13.1 - 17.8) | 8.8 (7.3 - 10.7) | 40.0 (36.9 - 43.3) | 39.2 (36.4 - 42.1) |
| Dominica | 17.3 (14.5 - 20.4) | 15.2 (12.9 - 17.8) | N/A | N/A |
| Dominican Republic | 16.3 (13.4 - 19.7) | 11.9 (9.7 - 14.5) | 59.2 (55.0 - 63.3) | 58.1 (54.3 - 61.7) |
| Ecuador | 31.7 (30.3 - 33.1) | 26.2 (24.7 - 27.7) | 32.8 (31.4 - 34.3) | 41.6 (39.9 - 43.3) |
| El Salvador | 17.4 (15.1 - 19.9) | 9.5 (7.7 - 11.7) | 65.0 (61.9 - 67.9) | 61.5 (58.1 - 64.8) |
| Grenada | 17.8 (14.9 - 21.0) | 12.6 (10.4 - 15.1) | 59.6 (55.6 - 63.5) | 59.6 (56.1 - 63.1) |
| Guatemala | 17.4 (15.7 - 19.1) | 10.8 (9.5 - 12.2) | 70.1 (68.0 - 72.2) | 64.8 (62.6 - 66.9) |
| Guyana | 18.1 (15.8 - 20.7) | 13.7 (11.9 - 15.7) | 64.1 (61.0 - 67.1) | 64.4 (61.7 - 67.0) |
| Honduras | 19.2 (16.6 - 22.1) | 12.4 (10.4 - 14.8) | 70.1 (66.8 - 73.2) | 70.3 (67.1 - 73.2) |
| Jamaica | 23.5 (20.4 - 26.8) | 21.6 (18.9 - 24.6) | 50.1 (46.3 - 54.0) | 38.7 (35.4 - 42.1) |
| Mexico | 37.8 (35.2 - 40.5) | 26.1 (23.9 - 28.5) | 26.8 (24.5 - 29.3) | 27.6 (25.4 - 30.0) |
| Montserrat | 22.7 (15.0 - 32.5) | 25.0 (17.4 - 34.4) | 48.9 (38.4 - 59.5) | 51.5 (41.5 - 61.3) |
| Paraguay | 23.1 (20.9 - 25.5) | 12.2 (10.6 - 14.0) | 68.2 (65.6 - 70.6) | 61.8 (59.3 - 64.3) |
| Peru | 16.5 (14.6 - 18.6) | 14.3 (12.6 - 16.3) | 70.7 (68.2 - 73.1) | 70.9 (68.4 - 73.2) |
| Saint Kitts and Nevis | 22.1 (19.2 - 25.3) | 14.8 (12.6 - 17.2) | 46.8 (43.1 - 50.5) | 35.3 (32.2 - 38.4) |
| Saint Lucia | 16.7 (13.6 - 20.2) | 14.4 (12.0 - 17.3) | 45.7 (41.4 - 50.1) | 47.8 (44.1 - 51.6) |
| St. Vincent and the Grenadines | 16.3 (13.4 - 19.6) | 11.6 (9.3 - 14.3) | 58.2 (53.9 - 62.3) | 61.4 (57.5 - 65.1) |
| Suriname | 22.3 (19.7 - 25.2) | 16.9 (14.6 - 19.4) | 55.1 (51.8 - 58.4) | 54.9 (51.7 - 58.1) |
| Trinidad and Tobago | 23.7 (21.7 - 25.8) | 15.6 (14.0 - 17.3) | 55.7 (53.3 - 58.2) | 45.6 (43.3 - 47.8) |
| Uruguay | 22.4 (20.4 - 24.6) | 9.0 (7.7 - 10.4) | 43.4 (41.0 - 45.9) | 37.2 (34.9 - 39.4) |

N/A: Not available.
